# Polarization-Dependent Gratings Based on Polymer-Dispersed Liquid Crystal Cells with In-Plane Switching Electrodes

**DOI:** 10.3390/polym14020297

**Published:** 2022-01-12

**Authors:** Chia-Yi Huang, Shih-Hung Lin

**Affiliations:** 1 Department of Applied Physics, Tunghai University, Taichung 40704, Taiwan; chiayihuang@thu.edu.tw; 2 Department of Optometry, Chung Shan Medical University, Taichung 40201, Taiwan; 3 Department of Ophthalmology, Chung Shan Medical University Hospital, Taichung 40201, Taiwan

**Keywords:** grating, polymer-dispersed liquid crystal, in-plane switching

## Abstract

A diffraction grating of polymer-dispersed liquid crystal (PDLC) with polarization-selective characteristics is investigated. Electrically controllable gratings are produced using In-Plane Switching (IPS) electrodes. Indium tin oxide (ITO) electrodes with a stripe pattern are used to generate a horizontal electric field parallel to the substrate on a single glass substrate. It is known from the experimental results that the number of diffraction orders can be controlled by applied voltage. Except for the zeroth order, the consistently highest intensity can be obtained for every other order of diffraction, and the polarization direction of the diffraction is perpendicular to the direction of the electrode stripes. The polarization direction of the zeroth order diffraction is parallel to the direction of the electrode stripes. Therefore, it can be used as a filter for light polarization.

## 1. Introduction

The combination of polymers with liquid crystals (LCs) has been widely investigated. Its property of compounding has aroused widespread attention. However, the LC material has been promoted and applied to displays. The birefringence of LC molecules and the electrically controllable alignment direction of LC molecules have enabled LC materials to be developed into various optical components, which are suitable for a wide range of electro-optical applications. The combination of polymer and LC can be divided into several applications depending on the properties of the material, such as polymer photoconductive film combined with LCs [1,2], LCs combined with polymer structures [3,4], polymer-stabilized LCs [5,6,7], polymer-dispersed LCs (PDLCs) [8,9,10,11,12,13,14,15,16,17,18,19], and LC elastomers [20]. PDLCs are composite materials that consist of LCs dispersed in a polymer. The LC–polymer mixture’s Ultraviolet (UV) light exposure can be used to separate the polymer from the mixture to reach phase separation. The prepolymer is photopolymerized to form the PDLCs. The matching of the refractive index between the LC domains and the polymer material will determine whether PDLCs produce light scattering. When the PDLC film is not controlled by an external force field, the refractive index mismatch between the polymer and the LC domains results in the light scattering of incident light. In previous research studies, electricity was used to control the orientation of LC molecules to be in the same direction, so that the refractive indexes between the LC domains and the polymer were matched, and the light scattering conditions disappeared to make the film transparent [8,9,10,11,12,13,14]. PDLC film is different from the traditional LC display. It achieves the display function with a light scattering effect. It is easy to make, flexible, and does not require additional polarizers, which can be used to make various optical devices with its electro-optic properties, such as displays [8,9,10,11,12], lenses [13,14], sensors [15,16,17], gratings [18,19], lasers [21,22,23], and light modulators [24,25,26].

Patterned electrodes can achieve periodic LC refractive index modulation, which enables a grating structure to be formed [27,28,29,30]. In this research report, it is shown that electrodes made with stripe patterns on a single glass substrate can be used in In-Plane Switching (IPS) technology to generate an electric field parallel to the glass substrate. This electric field was found to be able to control the direction of the LCs in the PDLC film, thereby forming a spatial periodic distribution between scattering and non-scattering, which resulted in the formation of gratings. This PDLC grating has the characteristic of selective polarization, and the polarization directions of the zeroth order diffraction and other order diffraction are perpendicular to each other. Under high voltage operation, it was possible to consistently obtain the highest diffraction intensity for each order of diffraction.

## 2. Materials and Methods

**Figure 1:**
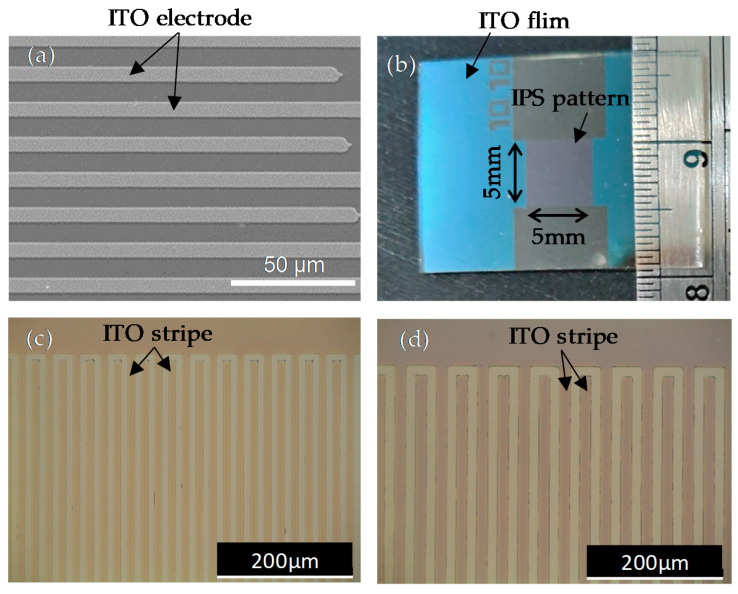
(**a**) The SEM image of the ITO conductive layer structure. (**b**) The image of ITO glass with an IPS pattern. Optical microscope images of designs of line width and space width of (**c**) 10 μm and 10 μm, and (**d**) of 15 μm and 15 μm.

This work used photolithography technology to make IPS electrode patterns on a glass substrate. To begin, a clean glass substrate with an area of 1.5 × 1.5 cm was prepared, and magnetron sputtering was used to coat the transparent conductive material, indium tin oxide (ITO, Gredmann Taiwan LTD., Taipei, Taiwan), on the surface of the glass substrate. The thickness of the ITO film was about 250 nm, and the surface resistivity was about 94 Ω/sq. The photoresist solution (ENPI202, Everlight Chemical Industrial Co., Taipei, Taiwan) was spin-coated on the ITO glass substrate. The ITO glass substrate with a photoresist layer was placed on the heating plate for soft baking. Under UV irradiation, the staggered spacing pattern of the stripe on the photomask was transferred onto the photoresist layer. A developer (az400k, Merck KGaA, Darmstadt, Germany) was used to develop the irradiated photoresist layer. An etching solution (LU-500, Tun-Hwa Electronic Material Co., Taichung, Taiwan) was used to remove the ITO film that was not covered by the photoresist, and then acetone was used to remove the remaining photoresist on the ITO glass substrate. Figure 1a shows a scanning electron microscope (SEM) image of the ITO conductive layer structure with the IPS pattern. Figure 1b shows that the IPS pattern area was 0.5 × 0.5 cm. Figure 1c,d show two optical microscope images that have two designs of line width and space width of 10 μm and 10 μm, and 15 μm and 15 μm, respectively. One ITO glass substrate with the IPS electrode structure and another clean glass substrate were paired to form an empty cell, and two plastic spacers of the same thickness were used as a cell gap. In this work, plastic spacers of three thicknesses were selected to produce cell gaps of approximately 5 μm, 10 μm, and 15 μm, respectively.

The materials used in this study to make PDLC are commercially available UV-curable prepolymer (NOA65, Norland Co., Jamesburg, NJ, USA) and nematic LC (NLC) (E7, Daily-Polymer Co., Kaohsiung, Taiwan). NOA65 is a UV-curing adhesive that produces good UV sensitivity when used with high concentrations of E7. The cured refractive index of NOA65 is n_p_ = 1.52. E7 has a birefringence that is based on an extraordinary refractive index n_e_ = 1.74 and an ordinary refractive index n_o_ = 1.52 at 25 °C. The refractive index mismatch between n_e_ = 1.74 and n_p_ = 1.52, and the refractive index matching n_o_ = 1.52 and n_p_ = 1.52 play an important role in the scattering of PDLCs and the optical phase of the grating. To prepare PDLC, the mixture was prepared consists of 30 wt.% NLC E7 and 70 wt.% polymer NOA65. The mixture was then shaken with an oscillator and was placed in the dark for 1 h to make it homogeneously mixed. We used a dropper to suck the mixture, inject the homogeneously mixed isotropic compound into the empty cell with the IPS electrode (made in advance through the capillary effect), and seal the edge of the cell with epoxy resin. Then, the cell, which was filled with the mixture, was exposed to an ambient temperature of 26 °C, and a non-polarized UV light with a wavelength of 365 nm and an intensity of 20 mW/cm^2^ was kept for a period of several minutes, which induced phase separation to form the PDLC film in the IPS electrode cells.

**Figure 2:**
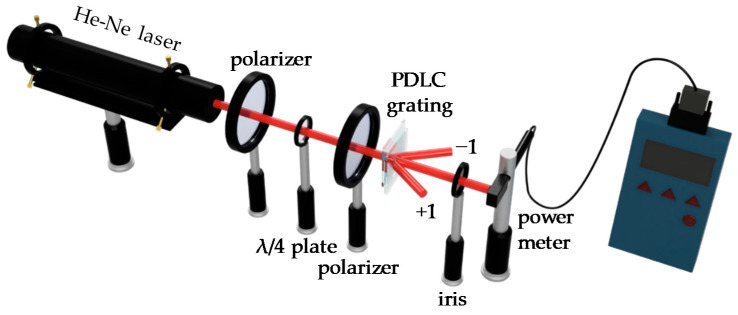
The measurement setup of the diffraction of PDLC grating.

Figure 2 shows the measurement setup of the diffraction signal of the PDLC grating using an unpolarized He-Ne laser beam (λ = 632.8 nm) as the probe beam. The probe beam was converted into a circularly polarized beam through a linear polarizer and a λ/4 plate. A polarizer was placed in front of the sample as an analyzer to select the direction of the linearly polarization of the incident beam. The PDLC cell with the IPS electrode was controlled to form PDLC grating by applying a power supply with 1 kHz AC. An iris and a power meter were placed in sequence behind the PDLC cell. The iris was used to select the diffraction order of the PDLC grating. The power meter (NOVA II, Ophir, Jerusalem, Israel) was used to detect the intensity of the diffraction of the PDLC grating.

## 3. Results and Discussion

**Figure 3:**
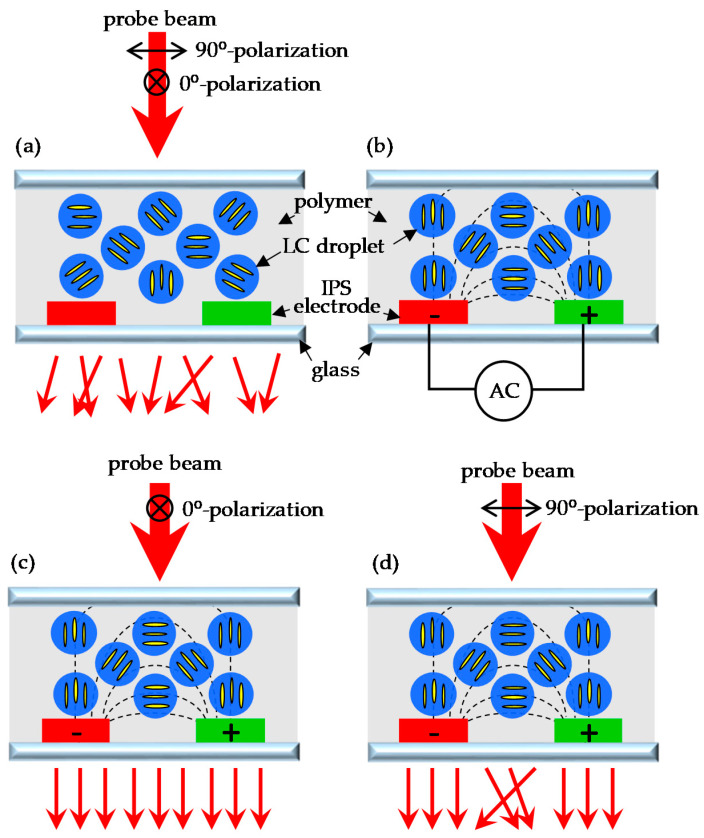
A schematic diagram and the operating principles of the PDLC grating (**a**) without voltage; (**b**) with voltage; (**c**) with probe beam of 0° polarization; (**d**) with probe beam of 90° polarization. The black dashed line represents the electric field.

Figure 3 shows a schematic diagram and the operating principles of the PDLC grating with the IPS electrode. In Figure 3a, it can be seen PDLCs existed between two glass substrates, and LC droplets or domains were randomly dispersed in the polymer. The direction of the molecules in the LC droplet was random. Since LC the directions were random, the light scattering of the PDLC cell was very strong when the refractive index was not matched. Due to the fact that LC molecules have positive dielectric anisotropy (Δε > 0), when an electric field was applied, the directions of the LC molecules tended to align with the electric field, which caused the LC directions in the droplet cavity to be reoriented by the electric field. The design of the IPS electrode produced a non-uniform electric field, causing the molecular direction of each LC droplet to be non-uniformly distributed with the electric field, as shown in Figure 3b. The electric field at the center of the electrode was perpendicular to the surface of the glass substrate, and the molecular direction of the LC droplets was also perpendicular to the surface of the substrate. Therefore, the 0° and 90° polarization of the incident beam were both subject to the LC ordinary refractive index (n_o_) in the center of the electrode, as shown in Figure 3c,d. Since the ordinary refractive index (n_o_) of the LC and the refractive index (n_p_) of the polymer matched each other, the PDLC at this position became transparent, and the light transmittance was greatly increased. On the other hand, the electric field at the center of the gap between the electrodes was parallel to the surface of the substrate, and the molecular direction of the LC droplets was also parallel to the surface of the substrate, so the 0° and 90° polarization of the incident beam were subject to different refractive indices. When 0° polarization of the incident beam was subject to an ordinary refractive index (n_o_) of the LCs, n_o_ matched the refractive index (n_p_) of the polymer, which caused the light transmittance to be increased, as shown in Figure 3c. When 90° polarization of incident beam was subject to the extraordinary refractive index (n_e_) of the LCs, n_e_ did not match the refractive index (n_p_) of the polymer, which caused a strong light scattering effect to be produced, as shown in Figure 3d. The refractive index of the LCs that were subject to 90° polarization from the electrode gap to the electrode center was between n_e_ and n_o_. When the PDLC cell generated spatially periodic scattering and non-scattering distributions, which resulted in a difference in the transmission intensity of the incident beam, the grating was formed. The refractive index of the LC that was subject to 0° polarization from the electrode gap to the electrode center was n_o_. Because it matched the refractive index (n_p_) of the polymer, the incident beam was directly transmitted without scattering, as shown in Figure 3c.

**Figure 4:**
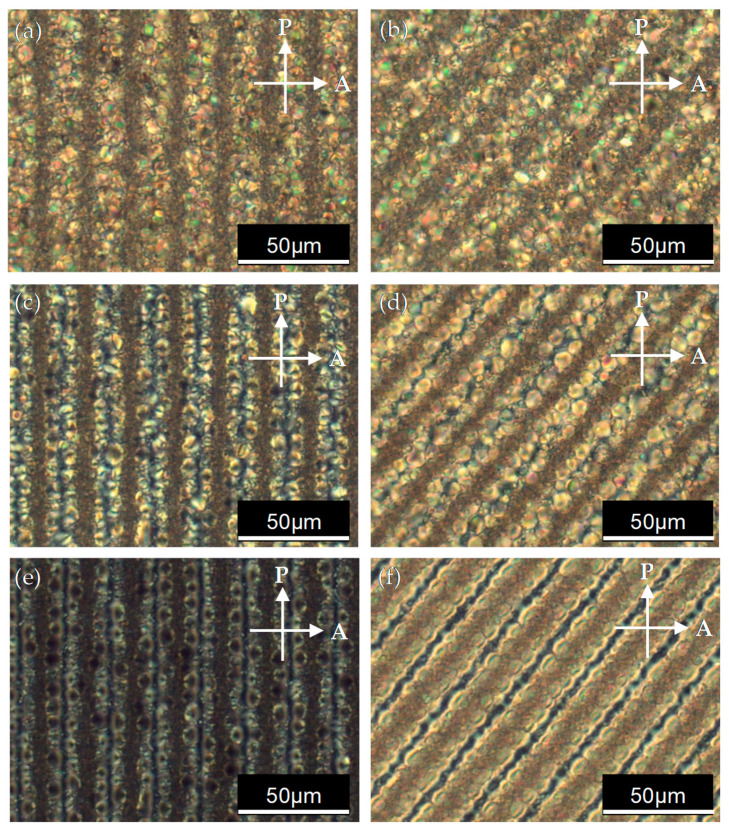
The POM images of the PDLC cell with the IPS electrode are shown at the applied voltage of (**a**,**b**) 0 V, (**c**,**d**) 50 V, and (**e**,**f**) 100 V. **P** and **A** denote the transmission axes of the polarizer and the analyzer, respectively. The direction of the IPS electrode stripes in the images on the left is parallel to the polarizer. The direction of the IPS electrode stripes on the right is at an angle of 45° with the polarizer.

Figure 4 shows the POM images of PDLC grating under voltage operation. Figure 4a,c,e show that the direction of the IPS electrode stripe was parallel to the polarization direction. Figure 4b,d,f show that the direction of the IPS electrode stripe and the direction of the polarizer were at an angle of 45°. Figure 4a,b show that the molecular orientation of the LC droplets was random when the IPS electrode had no applied voltage; thus, it can be observed that the LC droplets leaked light under the cross-polarized inspection. In Figure 4c,d, as the applied voltage reached 50 V, the direction of the LC realigned with the direction of the electric field. The direction of the electric field at the center of the ITO electrode was perpendicular to the glass substrate, and the direction of the LC was also perpendicular to the glass substrate. The probe light was only subject to the refractive index (n_o_) of the LC, so the center of the electrode stripe tended to darken under the cross-polarized inspection. Figure 4e,f show that as the applied voltage reached 100 V, the direction of the LC was aligned by the electric field generated by the IPS electrode. From the center of the electrode to the electrode gap, the direction of the LC tended to be parallel to the surface of the glass substrate. In Figure 4e, because the direction of the LC between the electrode center and the electrode gap was parallel to the direction of the analyzer, and the direction of the LC at the center of the electrode was perpendicular to the glass substrate, the PDLC cell was in a dark state under cross-polarized inspection. In Figure 4f, the direction of the LC between the center of the electrode and the electrode gap was at an angle of 45° with the direction of the polarizer, so this area leaked light and was bright.

**Figure 5:**
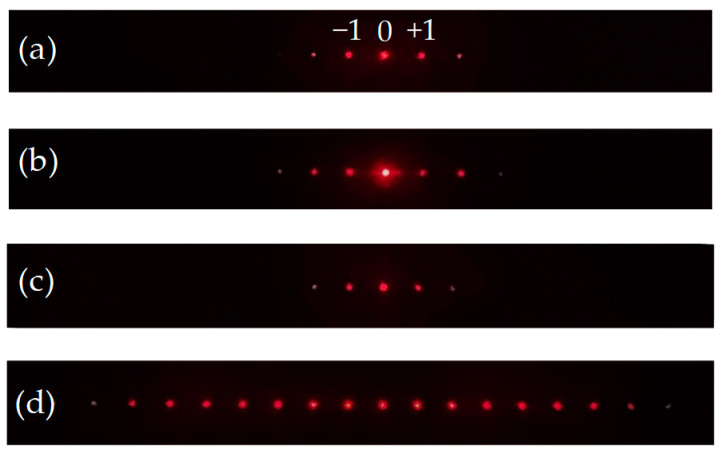
The diffraction patterns of the PDLC grating were probed using a He–Ne laser beam of (**a**) the 0° polarization: IPS without an applied voltage; (**b**) the 0° polarization: IPS with an applied voltage of 100 V; (**c**) the 90° polarization: IPS without an applied voltage; and (**d**) the 90° polarization: IPS with an applied voltage of 100 V.

Figure 5 shows the diffraction patterns of the PDLC grating probed using a He-Ne laser beam. Figure 5a shows the diffraction pattern of the PDLC cell probed by the incident beam of the 0° polarization when the IPS had no applied voltage. Without an external electric field, the molecular orientation of the LC droplets was random. Therefore, the diffraction pattern only came from the refractive index mismatch between the ITO electrode and the PDLC medium. Since the ITO layer was very thin, the diffraction effect generated was very weak. Figure 5b shows the diffraction pattern of the PDLC cell probed by the incident beam of the 0° polarization when the IPS electrode had an applied voltage of 100 V. Under the control of the electric field, the LC refractive index was subject to the incident beam of 0° polarization was n_o_ and matched the refractive index (n_p_) of the polymer. Therefore, the transmittance of the incident beam increased, and the intensity of the zeroth order diffraction increased greatly. However, the diffraction of other orders only came from the refractive index mismatch between the ITO electrode and the PDLC medium, so the diffraction intensity of other orders did not increase greatly as the transmittance of the PDLC increased. Figure 5c shows the diffraction pattern of the PDLC cell probed by the incident beam of the 90° polarization when the IPS electrode had no applied voltage. Without an external electric field, the diffraction pattern generated only came from the refractive index mismatch between the ITO electrode and the PDLC medium, so the diffraction effect generated was also very weak. Figure 5d shows the diffraction pattern of the PDLC cell probed by the incident beam of the 90° polarization when the IPS electrode had an applied voltage of 100 V. When the molecular orientation of LC droplets was aligned with the electric field distribution, the incident beam of the 90° polarization was able to undergo the change in the refractive index of the LC between n_o_ and n_e_. The spatial periodic change between scattering and transmission caused by the matching factor of the refractive index between LC and polymer resulted in the formation of grating. Therefore, when the incident beam of the 90° polarization was subject to the PDLC film, a strong diffraction effect was generated. Based on the above results, the following experiment used a probe beam of 90° polarization to measure the diffraction efficiency.

**Figure 6:**
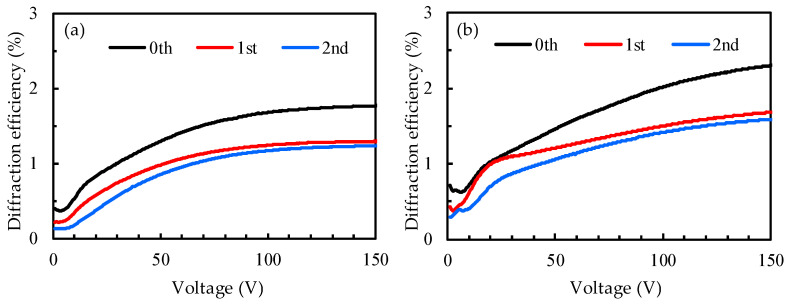
Diffraction efficiency of PDLC grating with an IPS electrode design of a line width and space width of (**a**) 15 μm and 15 μm, and of (**b**) 10 μm and 10 μm.

Figure 6 shows the diffraction efficiency of two PDLC gratings with different IPS designs with a probe beam of 90° polarization. The cell gaps of the two samples were both 15 μm. Figure 6a shows the experimental diffraction efficiency results with the IPS electrode width and spacing of 15 μm and 15 μm, respectively. When the voltage was increased to 100 V, the diffraction efficiency of the zeroth order reached the limit and the diffraction curve tended to be flat; moreover, the highest diffraction efficiency was 1.8% when the voltage was 150 V. The diffraction efficiency here is defined as the ratio of the transmitted intensity to the incident light intensity. As the voltage increased to 80 V, the first-order diffraction efficiency curve tended to be flat. The first-order diffraction efficiency at voltage = 150 V was 1.3%. The second-order diffraction efficiency also increased to a limit value as the voltage increased, and the diffraction efficiency curve became flat under high voltage. Figure 6b shows the experimental results of the diffraction efficiency with an IPS electrode width and spacing of 10 μm and 10 μm, respectively. The diffraction efficiency from the zeroth order to the second order became stronger as the voltage increased, and the diffraction efficiencies from the zeroth order to the second order, at a voltage of 150 V, were 2.3%, 1.7%, and 1.6%, respectively. The maximum applied voltage limit of the IPS electrode used in this experiment was 150 V. If this voltage was exceeded, the sample would have been destroyed. Therefore, the maximum applied voltage of all samples was only 150 V. The design of the IPS electrode could generate a horizontal electric field parallel to the surface of the glass substrate. When comparing the experimental results of Figure 6a,b, in the two IPS electrode designs, it can be seen that the experimental results of the electrode with a width and spacing of 10 μm and 10 μm were better. It is speculated that because the electrode spacing of 10 μm was relatively small, a relatively large lateral (horizontal) electric field could be generated at the same voltage, resulting in the better performance of LCs that were driven, and thus, the resulting diffraction efficiency could be relatively high. In addition, the sample thickness of the 15-μm cell gap shown in Figure 6 was too thick, so the lateral (horizontal) electric field generated by the IPS electrode could not drive the upper LC droplets of the sample, and thus, the orientation of the upper LC droplets of the sample was random. Therefore, the scattering of PDLC in the upper layer of the sample affected the diffraction performance of the grating.

**Figure 7:**
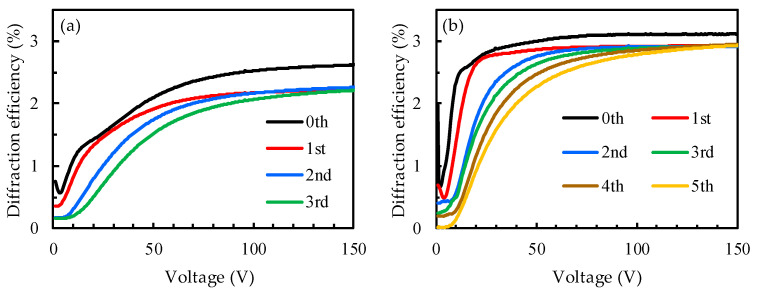
Diffraction efficiency of PDLC grating with cell gap of (**a**) 10 μm and (**b**) 5 μm.

The experimental result in Figure 6b proves that IPS electrode design with a line width and space width of 10 μm and 10 μm could obtain relatively high diffraction efficiency, but because the thickness of the sample used was relatively thick, the scattering generated by the PDLC on the cell upper layer affected the diffraction efficiency. Therefore, the experiment shown in Figure 7 involved the reduction in the thickness of the sample to test the diffraction efficiency of the PDLC grating. Figure 7a shows the diffraction efficiency of PDLC the grating with a cell gap of 10 μm. As the voltage increased to 150 V, except for the zeroth order, which had a diffraction efficiency of approximately 2.6%, the remaining three orders (first order to third order) all tended towards the same diffraction efficiency limit value of 2.2%. Compared with Figure 6b, the experimental results show that the diffraction efficiency was improved, which proves that the reduction in the cell gap was able to increase the diffraction efficiency. Figure 7b shows the diffraction efficiency of the PDLC grating with a smaller cell gap (5 μm). As the applied voltage increased, the diffraction order increased and the diffraction efficiency of all orders tended towards a maximum limit value. From the experimental results, it can be observed that the highest diffraction efficiency from the first order to the fifth order tended towards the same value, which was 2.9%. The highest diffraction efficiency of the zeroth order was about 3.0%. Under high voltage, the highest diffraction intensity from the first order to the fifth order was closer to the zeroth order. It is known from the experimental results that as the applied voltage increased, the number of diffraction orders also increased, and the highest diffraction intensity of each order was the same; thus, uniform diffraction performance could be obtained at high voltages.

**Figure 8:**
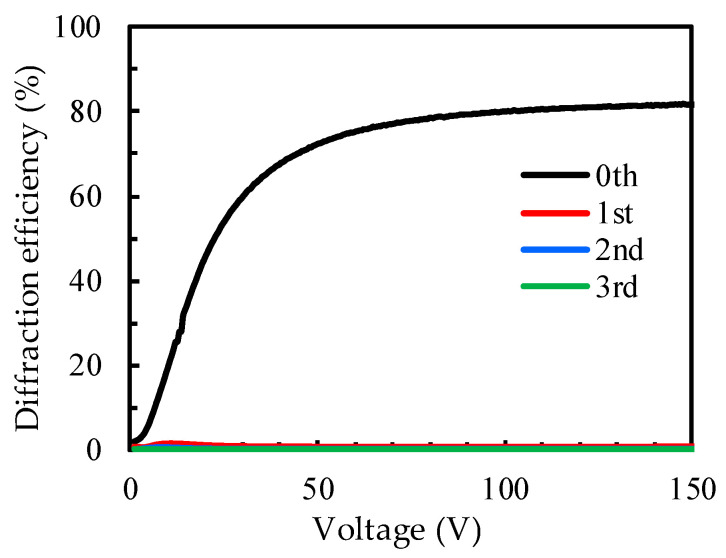
The diffraction efficiency of PDLC grating with a cell gap of 5 μm using an incident beam of 0° polarization.

Figure 8 shows the diffraction efficiency of the PDLC grating with a cell gap of 5 μm, as a function of voltage, using an incident beam of 0° polarization. It can be observed from the figure that the diffraction efficiency of the zeroth order increased significantly with the increase in voltage, and the highest diffraction efficiency was 81.8% at the applied voltage of 150 V. In contrast, the diffraction efficiency from the first to the third order did not increase significantly. This result conforms to the mechanistic principle described in Figure 3. When the voltage was applied to the PDLC cell with an IPS electrode, the incident light beam of 0° polarization was only subject to the refractive index (n_o_) of LC where n_o_ matched the refractive index (n_p_) of the polymer, and thus, the light transmittance could be increased. Therefore, the transmittance of the incident beam with 0° polarization increased strongly, and the diffraction intensity of the zeroth order also increased significantly. However, the diffraction of other orders involved only a weak diffraction signal generated by the mismatch of the refractive index of the PDLC and the ITO electrode, so there was no obvious diffraction effect due to the increase in the applied voltage.

**Figure 9:**
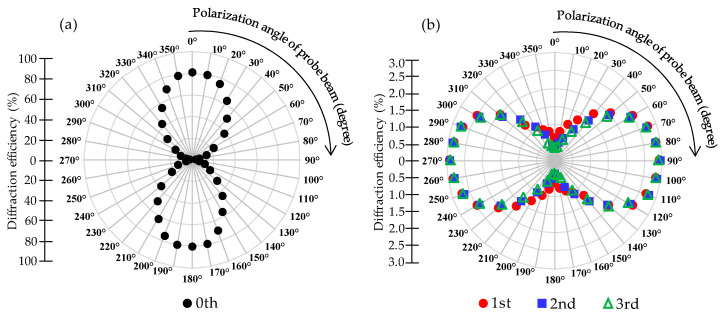
Polarization analysis of the diffraction of (**a**) the zeroth order and (**b**) the first to third order.

Figure 9 shows the polarization analysis of each order diffraction of the PDLC grating at an application voltage of 100 V. The cell gap of the sample used for this measurement was 5 μm and the applied voltage was fixed at 100 V. The second polarizer (analyzer) of the experimental setup shown in Figure 2 was rotated to change the polarization direction of the probe beam, and the polarization angle changes took every 10° as a unit. Every time the polarization direction of the probe beam was changed by 10°, the diffraction efficiency of each order was recorded. The polarization direction of the probe beam is indicated on the angular axis of the polar coordinates in Figure 9. The divergence axis of polar coordinates represents the efficiency of diffraction, recording the diffraction efficiency of each order. Figure 9a shows the measured diffraction efficiency of the zeroth order diffraction as the polarization angle of the probe beam changed. The highest diffraction efficiencies could be obtained in the directions of 0° and 180°, while relatively small diffraction efficiencies were obtained in the directions of 90° and 270°. The above results indicate that the zeroth order diffraction belonged to linear polarization, and the polarization direction was in the directions of 0° and 180°. Figure 9b shows that the diffraction efficiencies of the first order to the third order varied with the polarization direction of the probe beam. From the experimental results in the figure, it can be seen that the diffraction efficiencies of the first to third order diffraction in the directions of 90° and 270° were relatively high, while the diffraction efficiencies in the directions of 0° and 180° had minimal values. The results indicate that the first to third order diffraction belonged to linear polarization, and their polarization directions were in the directions of 90° and 270°. It is estimated that the polarization directions of other high-order diffractions were also 90° and 270°. When the applied electric field was turned on, there was a strong correlation between the diffraction intensity and the polarization direction of the probe beam. The polarization direction of the zeroth order diffraction was perpendicular to the polarization directions of the other orders.

## 4. Conclusions

In this study, a grating in PDLC film was successfully produced using IPS electrodes. The molecular direction of the LC droplets in the PDLC film could be realigned with the uneven electric field generated by the IPS electrode. By using the electric field to drive the PDLC grating, the increase or decrease in the diffraction order could be controlled, and each order of diffraction could reach a consistent intensity of diffraction. The zeroth order had a polarization of 0°, and the remaining orders had a polarization of 90°. Therefore, this PDLC grating was found to have the characteristics of polarization selection.

## Data Availability

The data presented in this study are available on request from the corresponding author.
